# Scutellaria barbata D.Don (SBD) extracts suppressed tumor growth, metastasis and angiogenesis in Prostate cancer via PI3K/Akt pathway

**DOI:** 10.1186/s12906-022-03587-0

**Published:** 2022-05-03

**Authors:** Dongya Sheng, Bei Zhao, Wenjing Zhu, Tiantian Wang, Yu Peng

**Affiliations:** 1 Yueyang Hospital of Integrated Traditional Chinese and Western Medicine, Shanghai University of Traditional Chinese Medicine, Shanghai, China; 2 Institute of Interdisciplinary Integrative Medicine Research, Shanghai University of Traditional Chinese Medicine, Shanghai, China

**Keywords:** Prostate cancer, Scutellaria barbata D. Don, PI3K/AKT, Metastasis, Apoptosis, Angiogenesis

## Abstract

**Background:**

Scutellaria barbata D.Don (SBD) is derived from the dried whole plant of Labiate which has been widely used to treat patients with multiple cancer. It was previously reported that the ethanol extract of SBD is able to promote apoptosis, and inhibit cell proliferation and angiogenesis in cancer.

**Materials and methods:**

CCK8, Edu assays and colony formation assay were performed to assess the effect of SBD on PCa cell growth. Effect of SBD on apoptosis and cell cycle was detected by flow cytometry. Transwell and wounding healing assay were conducted to detect the invasion and migration activities of PCa cells. Western blot was employed to detect the protein expression. 2RRV1 mouse xenograft model was established to detect the effect of SBD on prostate cancer. Angiogenesis was analysed by coculturing PCa cell lines and HUVECs.

**Results:**

The results showed that SBD induced a significant decrease in cell viability and clonogenic growth in a dose-dependent manner. SBD induced cell apoptosis and cell cycle G2/M phase arrest by inactivating PI3K/AKT signalling pathway. Treatment with SBD also significantly decreased the cell migration and invasion via phenotypic inversion of EMT that was characterized by the increased expression of E-cadherin and Vimentin, and decreased expression of N-cadherin, which could be partially attributed to inhibiting PI3K/AKT signalling pathway. Subsequently, using AKT inhibitor MK2206, we concluded that PI3K/AKT are also involved in cell apoptosis and metastasis of PCa cells stimulated by SBD. Apart from its direct effects on PCa cells, SBD also exhibited anti-angiogenic properties. SBD alone or conditioned media from SBD-treated PCa cells reduced HUVEC tube formation on Matrigel without affecting HUVEC viability. Furthermore, 22RV1 xenograft C57BL/6 mice treated with SBD in vivo showed a significant inhibitory in tumour size and tumour weight without toxicity. In addition, administration with medium- or high-dose of SBD significantly inhibited the cell proliferation and enhanced the damage to tumour tissues.

**Conclusions:**

Collectively, our in vitro and in vivo findings suggest that SBD has the potential to develop into a safe and potent alternative therapy for PCa patients.

**Supplementary Information:**

The online version contains supplementary material available at 10.1186/s12906-022-03587-0.

## Introduction

Prostate cancer (PCa) is a common male genitourinary tumour and poses threat to the health of men worldwide [1, 2]. It is a crucial health problem globally, which brings a huge economic burden to the society [3]. Despite conventional therapeutic approaches aimed to prolong progression-free survival, patients diagnosed with PCa are still at risks of recurrences, low postoperative survival rates, high metastasis rate and harmful side-effects that led to resistance [4].

The epithelial-to-mesenchymal transition (EMT) is considered a hallmark of the aggressive invasion and metastasis of advanced tumours [2]. In previous studies, EMT can bring about the malignant transformation of PCa, thus leading to its invasion and migration [5].

Tumour angiogenesis, as a significant cancer hallmark, plays a crucial role in tumour progression, including rapid growth, recrudesce and metastasis [6, 7]. Through excessive secretion of pro-angiogenic factor, tumour cell continuously activate endothelial cells to ‘sprout’ in the original blood vessels to form new vascular structures [8]. At the same time, angiogenesis supplies the fundamental nutrients and oxygen to cancer cells, and also act as a way for metastasis [9]. Therefore, inhibiting tumour angiogenesis has been an effective strategy for cancer therapy since decades ago.

The application of traditional Chinese medicine (TCM) in cancer treatment has been recorded in Chinese medical texts and pubications for more than two millennium. Currently, herbal medicine is widely used in the treatment of PCa patients due to its safety and clinical efficacy, in particular its synergistic effects and fewer side effects and lower toxicity [10, 11]. In many cancer centers around the world, Chinese medicine is often widely combined with Western medicine in complementary and alternative ways to create synergistic effects and improve efficacy. SBD has been known in traditional chinese medicine for over 400 years for its heating-releasing and detoxifying proprties, which means it can kill aberrant cells in the human body. Mounting evidence showed that SBD had an inhibitory effect on lung cancer, liver cancer, leukemia, and breast cancer [5, 12–14]. At the same time, it has been reported that Hedyotis diffusa Willd (HD) had positive effect on the treatment of malignant tumor [15, 16]. These studies have been indicated that SBD is a potential drug in anti-tumor treatment. Nevertheless, the underlying molecular mechanism of SBD in PCa and cancer metastasis has not yet been elucidated.

In the present study, we investigated the anti-tumor, anti-metastasis activities of SBD and its mechanism by using a series of *in vitro* assays and *in vivo* mouse model. Subsequently, we investigated the antiangiogenic effect of SBD on human umbilical vein endothelial cell (HUVEC). This study will offer a new clue to the clinical application of SBD.

## Materials and methods

### Preparation of SBD

The raw herb was purchased by Huayu pharmacy company in Shanghai. (Lot:210526; Place of production: Shanxi). And they were identified by Liuhong, from the department of pharmacology, Yueyang Hospital of integrated Traditional Chinese and Western Medicine. Preparation of SBD was established as previously described. Briefly, the dried rhizomes of SBD were ground into powder, which was then extracted with double-distilled water by reflux extraction for 1.5 h/time 2times. Ninety-five percent ethanol was added to the mixed extract to lower the final concentration of ethanol to 85% (v/v). The precipitated polysaccharide component was taken out by the filtration device. A rotary evaporator concentrated the resulting solution at 50 °C under condition of lower pressure. Eventually, the extract was dissolved again in methanol for carrying out liquid chromatography (HPLC) analysis [17].

### Analysis of SBD

SBD was analysed on uadrupole time-of-flight mass spectrometry (UPLC-Q- TOF-MS). The system was completed on a Waters ACQUITY UPLC HSS C18 column and the column temperature was 25 °C. Mass spectrometry analysis was conducted on an AB Sciex Triple TOF® 4600 system equipped with an ESI source. The detection wavelength was measured at 254 nm.

### Cell culture and reagents

Human umbilical vein endothelia cells (HUVECs), human PCa cell lines (PC-3 and DU145), normal prostate cells (wpe-int) and Mouse PCa cell line (22RV1) were purchased by Chinese Academy of Sciences, China (Shanghai, China) and were cultured in DMEM supplemented 10% fatal bovine serum (FBS) and 1% penicillin-streptomycin at 37 °C and 5% CO_2_. Cells were treated with SBD in fresh medium at different concentration, and the control group employed an equal volume of DMSO.

### Cell viability

Cells were exposed to stepwise increasing concentrations of SBD. After treatment with different concentration of SBD, the cell viability assay was detected using a CCK-8 kit (APE-BIO, USA) based on the manufacturer’ s instruction. Briefly, after 24 h or 48 h, OD value was measured by using CCK8 regent. The cell viability rate calculation formula is: Cell viability (%) = OD value of the experimental/OD value of the control group × 100%.

### Cell apoptosis assay

After 48 h of SBD treatment, PCa cells were harvested. Cell apoptosis was determined by an Annexin V-FITC/PI Apoptosis Detection Kit (Biolegend, USA, LOT: B324951). All results were analysed by flow cytometer (Beckman Coulter, MA, USA).

### Cell clonogenicity assay

Cells were divided into three group: a control group, a 50 μg/mL SBD and a 100 μg/mL SBD and maintained at the SBD containing completed medium 10 d. Subsequently, the cells were fixed by 4% methanol for 20 min, then stained with crystal violet for 20 min. Glacial acetic acid was added into plate, subsequently the OD value of each well at 590 nm was measured by microplate reader.

### Cell cycle analysis

Cells were stimulated with SBD (50 and 100 μg/mL) for 48 h, and then fixed by 70% ethanol at −20 °C overnight. Afterwards, cells were washed with phosphate-buffered saline (PBS), and incubated with 500 uL of propidium iodide (PI) (BD Phamingen, USA) staining solution for 15 min. Afterwards, cells were detected under a FACS-calibur flow cytometry (Beckman-Coulter, China).

### Cell proliferation analysis

Cell proliferation was measured using EDU solution (Beyotime, Shanghai, China, LOT: 10181900522) for 2 h, and then stained with 1 × Hoechst 33342 solution (Beyotime, Shanghai, China, LOT:H3117050) for 10 min at 25 °C. Morphological changes were observed using a fluorescence microscope. (Magnification, ×100).

### Wound healing assay

The cells in each well were linearly scraped by a 10 μL of sterile pipette tip. Afterwards, each well was added fresh medium with SBD, and incubated for 48 h. The images of cross were photographed under the microscope.

### Tube formation assay

PCa cells were cultured, and treated without or with SBD or phorbol 12-myristate 13-acetate (PMA) at the different concentration for 48 h. The conditioned media (CM) was collected and applied to HUVEC. Matrigel matrix gel (50 μL/well) was added into a 96-well plate to incubate for 30 min. Subsequently, 1 × 10^4^ HUVECs were dispersed in the CM and then seeded on the Matrigel layer for incubation at 37 °C in 5% CO_2_ for 4 h. Then photography was performed with a microscope.

### Cell migration and invasion assays

For the cell invasion assay, chambers were pre-coated with Matrigel (BD Biosciences, San Jose, CA). Cells stably transfected with different constructs were starved overnight, and then seeded in the upper chamber (Corning, Billerica, MA) at a density of 1 × 10^5^ cells/mL in 200 μL of medium without FBS. Meanwhile, lower chamber plated into 600 μL of medium with 15 mL FBS. 24 h later, when the incubation was finished, a cotton swab was used to clear non- migrating (or non- invading) cells in the upper chamber. We quantified invading cells by using manual counting.

### Western blotting assay

Cells and tumour tissue were harvested and lysed with RIPA buffer. Proteins were transferred to polyvinylidene fluoride (PVDF) membranes for 90 min. In this study, primary antibodies were used as follow: Cleaved-caspase-3 (LOT:3), Bax (LOT:12), Bcl-2 (LOT:5), p-AKT (LOT:21), AKT (LOT:9), p-PI3K (LOT: 12), PI3K (LOT:6), E-cadherin (LOT:11), N-cadherin (LOT:13) and Vimentin (LOT: 10) (Cell Signalling Technology, USA). The blots were cut prior to hybridisation with antibodies during blotting. After washing, the secondary antibodies were used to visualize the blot under the Bio-Rad imaging system.

### Tumor xenograft in mice model

Male C57BL/6 mice from the Shanghai University of traditional Chinese Medicine Laboratory Animal Management Department. All mice were conducted according to the Guide for the Care and Use of Laboratory Animals of Shanghai University of traditional Chinese Medicine Laboratory Animal Management Department, as approved by the Animal care Committee of Shanghai Province, China (Approval No. P2SHUTCM210715001). The mice were subcutaneously engrafted in the right-hind flank with 1 × 10^6^ 22RV1 cells (day 0). Mice were randomly allocated to 4 groups (n = 5), including the control group (0.9% saline), SBD medium-dose (50 mg/kg), SBD high-dose group (100 mg/kg). SBD was given orally once a day. The body weight and tumour size of the mice were monitored every 2 days. Tumour volumes (V) were calculated using the formula V = (L × D^2^)/2, where L is the greatest diameter and D is the smallest diameter of the tumour. During the experiment, the mice did not show any discomfort. After 16 d, mice sacrificed via cervical dislocation, than the tumours were extracted to be weighed. All sections of this research adhere to the ARRIVE Guidelines for reporting animal research.

### Histological analysis

Tumour tissue were perfused with 4% paraformaldehyde before embedded in paraffin and sectioned at a thickness of 3 μm thick. For H&E staining, the tissue was sliced ​​after deparaffinization, stained and fixed on a glass slide with neutral gum. Tumour sections were immunohistochemically stained with Ki67, E-cad, N-cad, and Vim antibodies according to the steps of manufacturer. After the above steps were completed, photos were taken using the microscope.

### Statistical analysis

All data of more than 3 independent experiments are presented as the Mean ± Standard Deviation. Unpaired Student’s t-test was used for two experimental groups and one-way ANOVA test was used for multiple groups. A two-tailed value of *p* < 0.05 was considered statistically significant. Statistics were done using GraphPad Prism 8.0.

## Results

### The chemical components of SBD

**Fig. 1:**
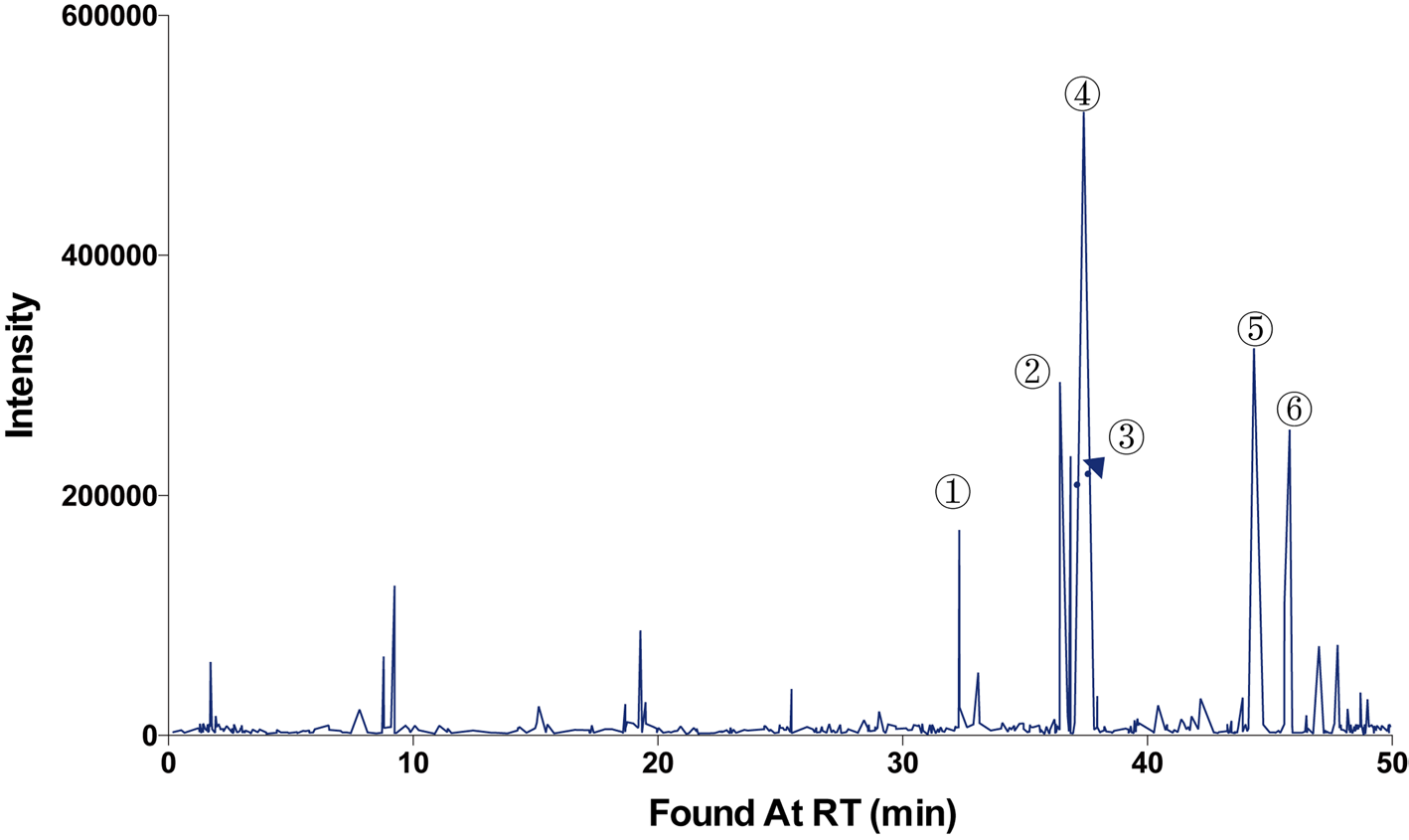
Total ion chromatograph of SBD in positive ion modes. High-performance liquid chromatography (HPLC) analysis of SBD and control sample. There were four peaks in HPLC, which were identified as ① apigenin, ②scutellarin, ③ baicalein, ④ luteolin, ⑤naringenin, ⑥wogonin

The related chromatograms were shown in Fig. 1.

### SBD inhibited cell proliferation and induced cell cycle arrest in PCa cells

**Fig. 2:**
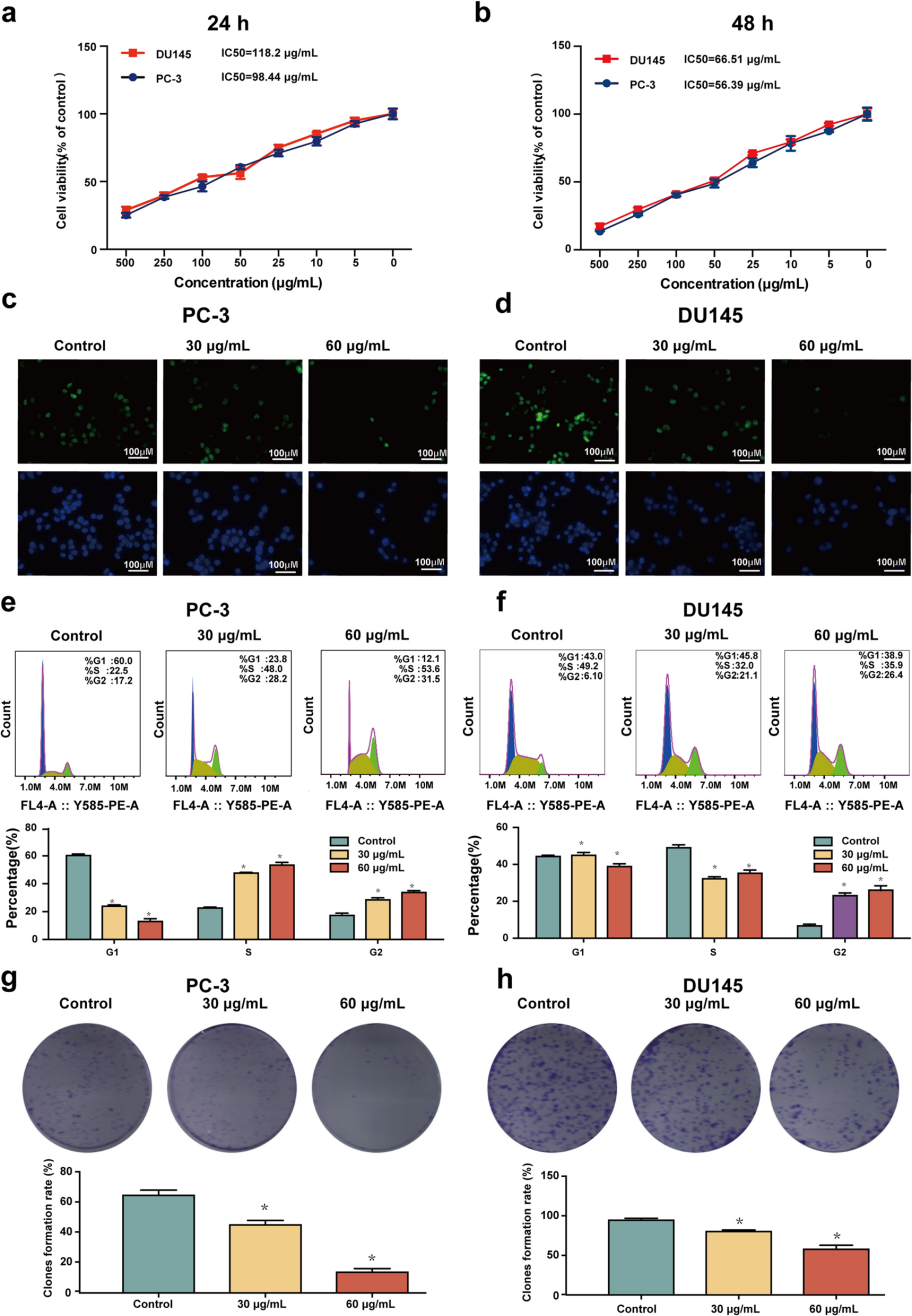
SBD inhibits the proliferation of PCa cells. Cell viabilities were measured by CCK-8 assay after treatment with indicated concentrations of SBD for 24 h (**a**) and 48 h (**b**). The cell proliferation and apoptosis of PC-3 (**c**) and DU145 (**d**) cell lines with or without SBD treatment determine by EdU and Hoechst staining. Cell cycle of PC-3 (**e**) and DU145 (**f**) cell lines measured by flow cytometry analysis after SBD treatment for 48 h. The colony formation of PC-3 (**g**) and DU145 (**h**) cell lines with or without SBD treatment. Date was mean ± SEM (n = 4), *p* < 0.05 (*)

The cytotoxicity of SBD on PC-3 and DU145 cell lines was detected by CCK8 assay. Cells were exposed to various concentration of SBD for 24 h (Fig. 2a) and 48 h (Fig. 2b). According to the results of cell viability, SBD showed an obvious cytotoxic effect against PC-3 and DU145 cell lines in a time and concentration dependent manner, with IC_50_ of 98.44 μg/mL and 118.2 μg/mL, in 24 h respectively, and 56.39 μg/mL and 66.51 μg/mL in 48 h, respectively. SBD had marginal anti-tumour effects on PC-3 than on DU145. At the same time, we detected the cell viability of SBD on normal prostate cells (wpe-int). There was no obvious difference between the control group and the SBD treatment (Fig. S1).

Afterwards, the cytotoxic phenotypes in PC-3 (Fig. 2c) and DU145 cell lines **(**Fig. 2d**)** were detected under EdU incorporation staining. The observations demonstrated that SBD concentration-dependently decreased the radio of EdU-positive nuclei in both PCa cell lines.

Immortal proliferation is a crucial feature of tumour and timing of proliferation relies on the transition speed of cell cycle [18]. It has been observed that cell cycle plays an important role in the progression of PCa cells [19]. The effect of SBD on cell cycle of PC-3 (Fig. 2e) and DU145 cell lines (Fig. 2f) was detected. The data demonstrated that most of the cells were arrested in the S and G2 phases after treatment with SBD in PC-3 cells. Except for an obvious increase in S and G2 phases cell population, SBD treatment decreased the G1 phase cell population (*p* < 0.05). Consistent with PC3 cells, treatment with SBD resulted in an apparent increase in the G2 phase cell population in the DU145 cell (*p* < 0.05). In addition, the colony formation experiment indicated that SBD suppressed the colony formation of PCa cells (Fig. 2g & h).

Taken together, the inhibitory effect of SBD on the cell proliferation and colony formation might be partially due to the cell cycle arrest at G2/M phase.

### The PI3K/AKT signaling pathway participates in SBD-induced PCa cell apoptosis

**Fig. 3:**
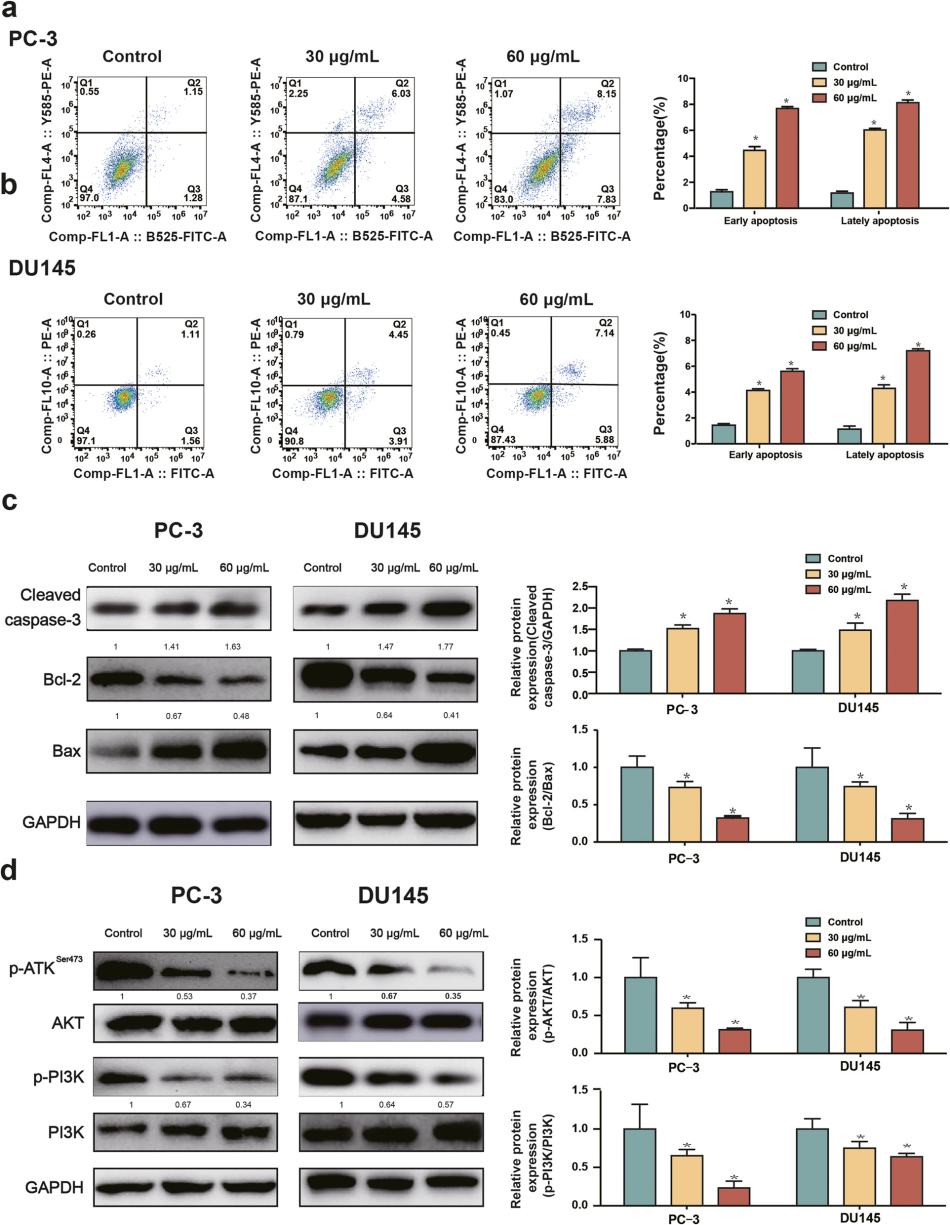
SBD inhibited PCa cell apoptosis through Akt/PI3K signalling pathway. Cell apoptosis measured by flow cytometry after SBD treatment with different concentrations for 48 h in PC-3 (**a**) and DU145 (**b**) cell lines; Protein expression levels of Cleaved caspase-3, Bcl-2 and Bax in PC-3 and DU145 cells determined by western blotting assay; (**c **and** d**) Relative protein expression levels of p-Akt, Akt, p-PI3K and PI3K with or without SBD treatment determined by western blotting assay. Date was mean ± SEM, *p* < 0.05 (*)

Increasing apoptosis is the main index to assess the efficacy of antitumor drugs [20]. We then investigated whether SBD has the ability to induce apoptosis the biological function of SBD in apoptosis, the cell apoptosis in SBD - treated PC-3 (Fig. 3a) and DU145 cells (Fig. 3b) was analysed with flow cytometry. The percentage of apoptosis cells was dramatically increased in PC3 and DU145 cells treated with SBD when compared with their controls (*p* < 0.05).

Thus, from prior reports, we hypothesized that SBD could constrain the expression of a panel of proteins associated with cellular apoptosis. The Western blot results was that SBD treated PCa cells boosted expression of activated Caspase-3, proving that apoptosis was the major mechanism of SBD -induced growth inhibition in PCa cells. After that, we examined the impact of SBD on the Bcl-2 family of proteins involved in apoptosis. There was an increase in the expression of pro-apoptotic Bax protein in both PC3 and DU145 cells treated with SBD. In addition, treatment with SBD for 48 h down-regulated the expression of anti-apoptotic Bcl-2 protein in PCa cells (Fig. 3c).

The abnormal activation of PI3K/AKT signalling pathway is very frequently observed in PCa. Furthermore, SBD significantly inhibited the phosphorylation of AKT and PI3K in PCa cells (Fig. 3d), which are consistent with the roles of PI3K/AKT signalling pathway in the regulation of cell apoptosis. These dates demonstrated that SBD promote PCa cells apoptosis via inactivation of PI3K/AKT signalling pathway.

### SBD inhibits migration, invasion, EMT progression and TME-angiogenesis

**Fig. 4:**
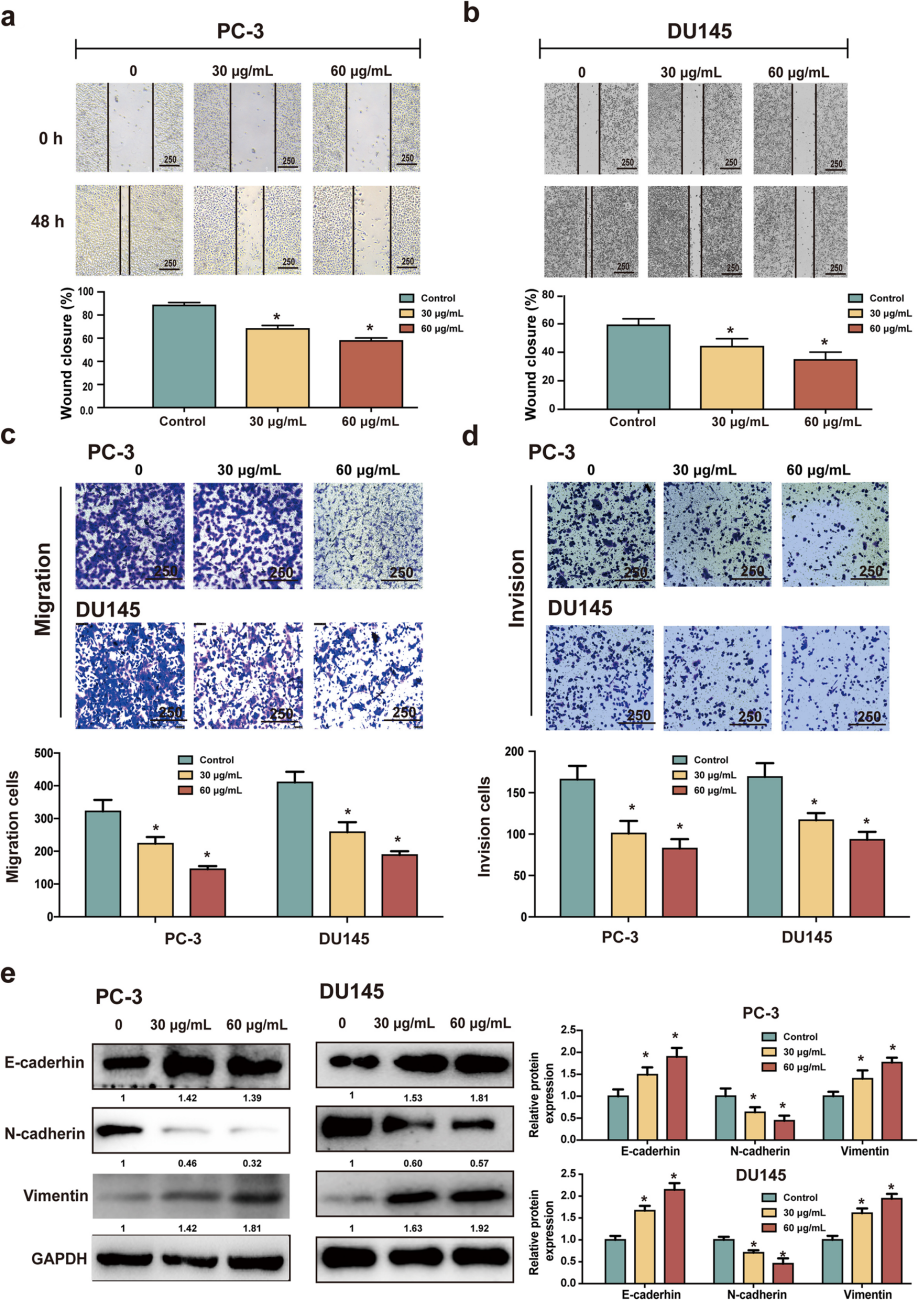
Effects of SBD on the migration and invasion capabilities in PC3 and DU145 cells. Wound-healing assay was conducted to examine cellular migration of PC-3 (**a**) and DU145 (**b)** at 0 and 48 h; Cell migration (**c**) and invasion (**d**) of PC3 and DU145 cells that underwent SBD treatment was determined by transwell assay; (**e**) The expression of EMT-related proteins in PC3 and DU145 cells with or without SBD treatment was determined by western blotting assay. Date was mean ± SEM, *p* < 0.05 (*)

The metastasis and invasion are closely related to the malignant degree and prognosis of the tumour [21]. According to the wound-healing assay, SBD significantly inhibited tumour cell migration in both PC3 (Fig. 4a) and DU145 cells (Fig. 4b) in a dose-dependent manner. To determine if SBD could supress migration and invasion of PCa cells, transwell assays were employed in PC3 and DU145 cells. As expected, treatment with SBD for 48 h reduced the number of invaded cells remarkably (*p* < 0.05, Fig. 4c & d). These results indicated that SBD decelerate PCa cells migration and invasion.

As reported in previous studies, EMT is inseparably associated with metastasis of tumour. To further study the potential mechanism of SBD on its anti-metastatic effect, we next assessed the expression of EMT-related protein though western blot. As indicated in Fig. 4e, the expression of the epithelial marker E-cadherin and Vimentin was significantly up-regulated by SBD, while the expression of N-cadherin was down-regulated remarkably in the SBD -treated cells compared to the control cells (*p* < 0.05, Fig. 4e). Collectively, these results showed that SBD can affect cancer cell invasion, migration and EMT-related protein.

### Blockade of AKT resulted in inhibition of SBD-mediated cell proliferation and migration

**Fig. 5:**
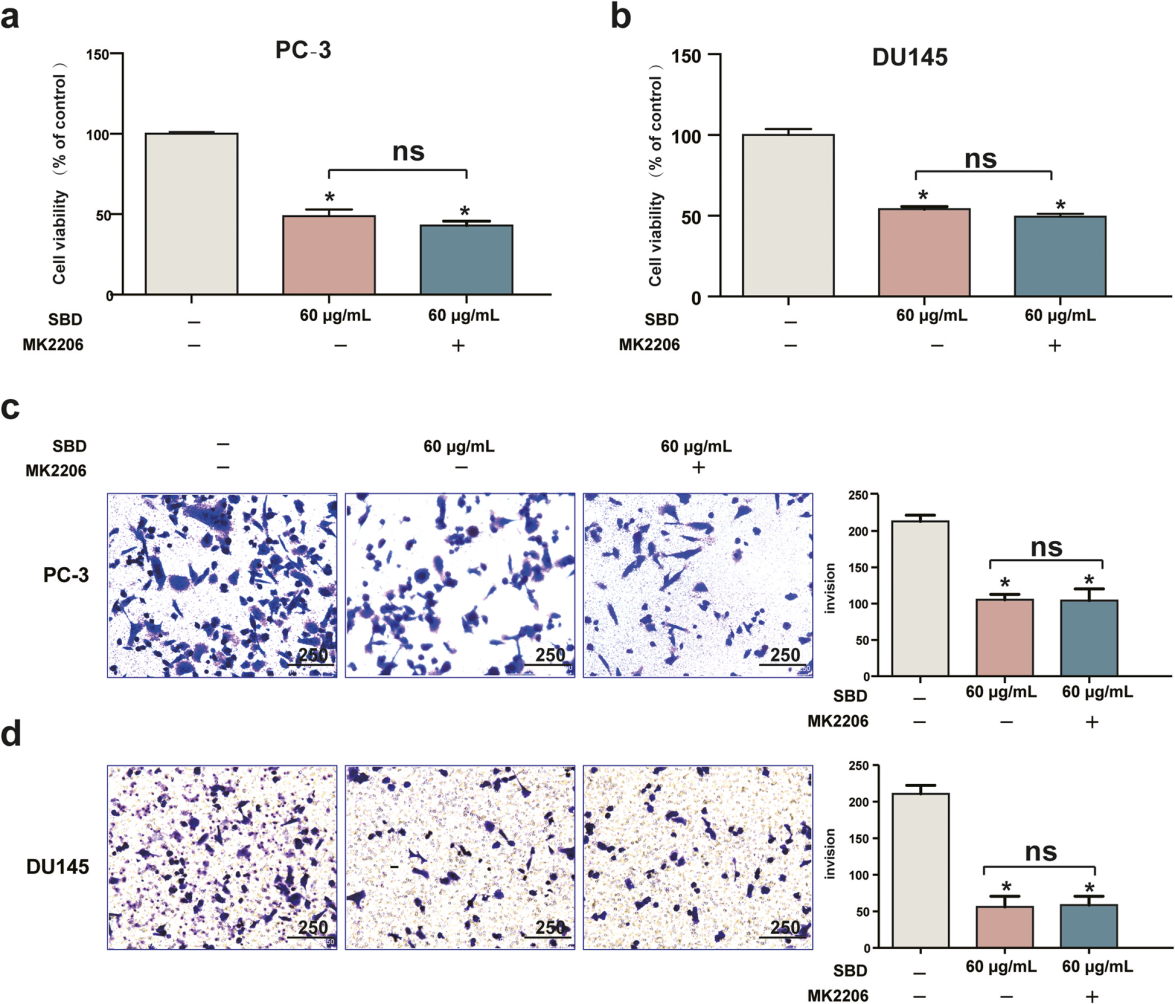
SBD inhibited PCa cell vitality and invasion target Akt. Cell viabilities of PC-3 (**a**) and DU145 (**b**) cell lines pre-treated by MK2206 were measured by CCK-8 assay after treatment with indicated concentrations of SBD. Cell invasion of PC-1 (**c**) and DU145 (**d**) cell lines that underwent SBD treatment was determined by transwell assay. Date was mean ± SEM (*n* = 4), *p* < 0.05 (*)

To further determine the involvement of the PI3K/AKT pathway in the inhibition of tumour cell growth by SBD, PC-3 and DU145 cells were pre-treated with 10 μM MK2206 (AKT-specific inhibitor) for 6 h, followed by 48 h of SBD treatment. After 48 h, cell viability was analysed using CCK-8 assay. Of note, co-treatment with SBD had no significant effect on cell viability compared with those treated alone with SBD (50 μg/mL) in PC-3 (Fig. 5a) and DU145 cells (Fig. 5b). In addition, co-treatment with SBD did not further decrease the expression of p-AKT (*p* > 0.05).

To further investigate if the cell invasion inhibitory effect of SBD is dependent on AKT/PI3K signalling pathway, MK2206 was pre-treated on PCa cells. The results showed that PC-3 (Fig. 5c) and DU145 cells (Fig. 5d) migration had no significant difference when compared with SBD (50 μg/mL) treatment alone (*p* > 0.05). These results above indicated that SBD inhibited cell viability and invasion in a mechanism dependent on the AKT/PI3K pathway partially.

### SBD inhibited EMT via AKT/PI3K pathway signaling

**Fig. 6:**
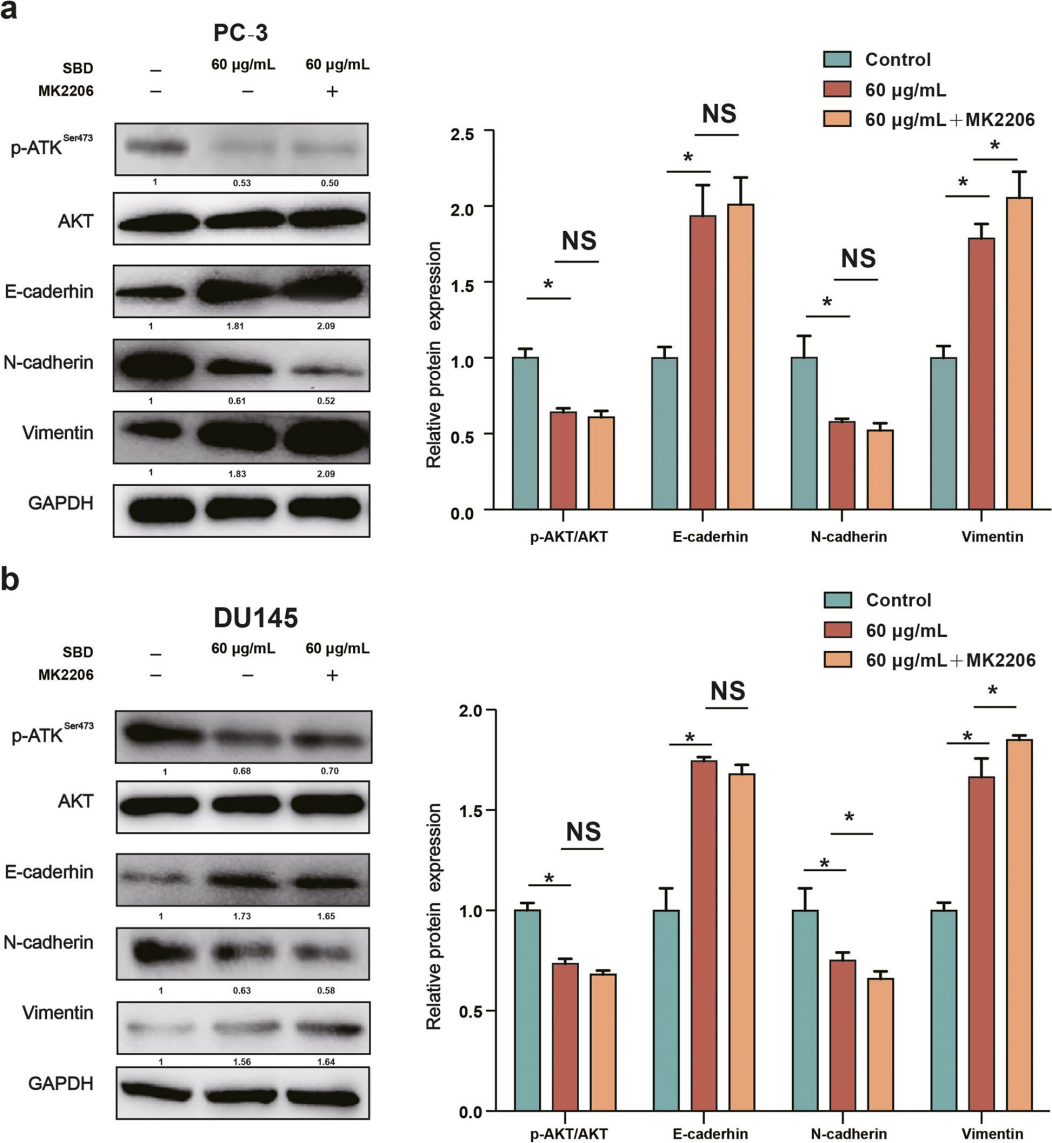
SBD inhibited PCa cell EMT-related protein target Akt. The relative protein expression levels of p-Akt, Akt, and EMT-related proteins in PC-3 (**a**) and DU145 (**b**) cell lines with or without SBD or MK2206 treatment was determined by western blotting assay. Date was mean ± SEM (*n* = 4), *p* < 0.05 (*)

Based on the abovementioned results, SBD may regulate EMT through PI3K/AKT signaling pathway. According to the results of western blot, we found MK2206 reversed effects of SBD on expression of p-AKT, N-cadherin, E-cadherin and Vimentin on PC-3 and DU145 cells (*p* < 0.05)(Fig. 6a & b). Collectively, these results suggested that treatment with SBD may partially inactivate PI3K/AKT signalling pathway, thereby inhibiting the cell proliferation and invasion, as well as preventing PCa cells from EMT progression.

### SBD suppressed tumor growth in 22RV1 cell xenograft mice model

**Fig. 7:**
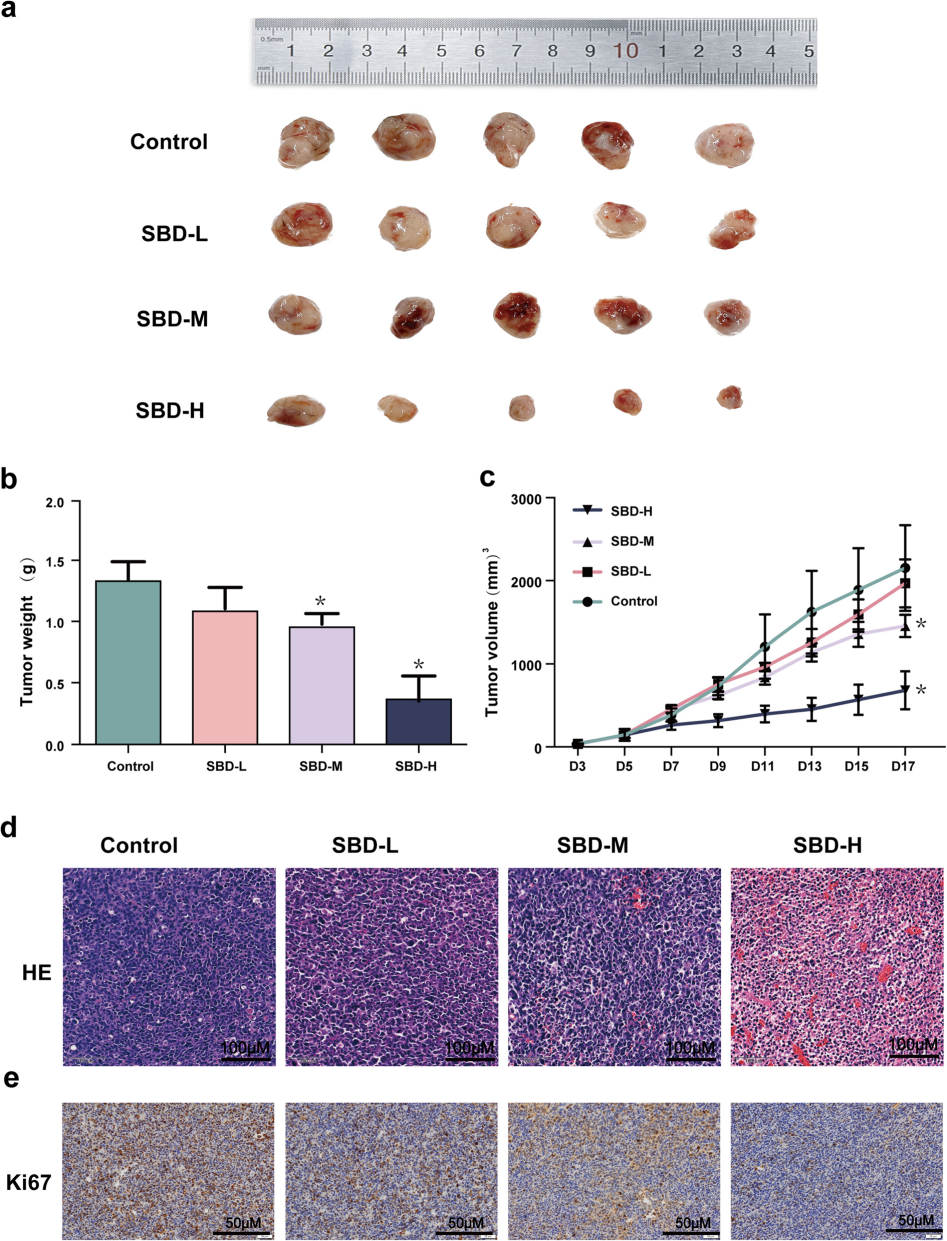
SBD inhibited tumour growth in xenograft mice model. **a** Xenograft tumours imaging of 22RV1 in different groups at the end of experiments; **b** Administration of different concentration SBD decreased the tumour weight (g); **c** Administration of different concentration SBD decreased the tumour volume (mm^3^); **d** Administration of different concentration SBD attenuated the tumour tissue damage measured by H&E staining; **e** The expression of Ki67 measured by IHC staining in tumour tissues from xenograft C57BL/6 mice Date was mean ± SEM (*n* = 5), *p* < 0.05

Next, to investigate the anti-tumour effect of SBD on the inhibition of PCa *in vivo*, we established 2RRV1 subcutaneous xenografts. We explored the effects of Low-dose (SBD-L), Medium-dose (SBD -M), and High-dose (SBD -H) of SBD treatment on tumour growth. As shown in Fig. 7a, b & c, administration of SBD in 22RV1-bearing mice markedly inhibited tumour volume and the tumour weight. Our results showed that there are significant differences between the SBD treatment and the control group. H&E and proliferating cell-related antigen Ki67 staining were conducted to explore the growth level of the xenografts. Ki67-positive rate was decreased markedly in the SBD-treated group with medium and high dose (Fig. 7d & e), which confirmed that subcutaneous tumour proliferation was significantly reduced in the SBD-M and SBD-L treatment group.

### SBD inhibited AKT/PI3K and EMT progression *in vivo*

**Fig. 8:**
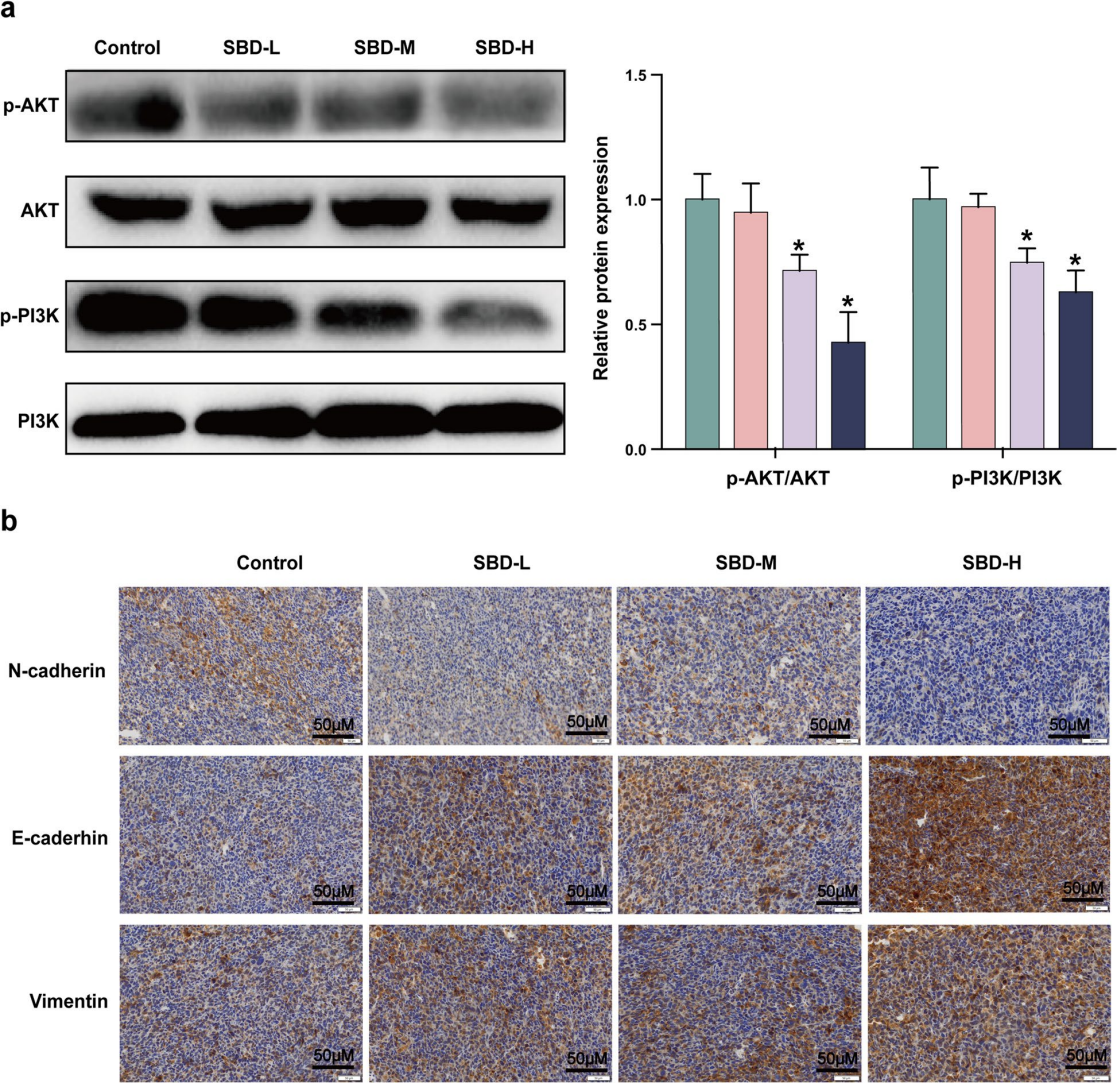
SBD inhibited EMT through Akt/PI3K signalling pathway in xenograft mice model. **a** The relative protein expression levels of p-Akt, Akt, p-PI3K and PI3K in xenograft tumours determined by western blotting assay; **b** The EMT-related proteins in xenograft tumours determined by IHC staining Date was mean ± SEM (*n* = 3), *p* < 0.05 (*)

Based on the results of the *in vitro* study, western blotting and immunohistochemical (IHC) assays were used to detect the expression levels of EMT-related proteins in tumour tissues. As shown in Fig. 8a, administration with SBD-M and SBD-H reduced the phosphorylation levels of PI3K and AKT remarkably (*p* < 0.05) in tumour tissues. In addition, according to the IHC staining results, the expression of E-cadherin and Vimentin was significantly up-regulated, while the expression of N-cadherin was down-regulated remarkably in SBD-M and SBD -H groups when compared to the control groups (*p* < 0.05, Fig. 8b).

### SBD blocks tube formation of human umbilical vein endothelial cells

**Fig. 9:**
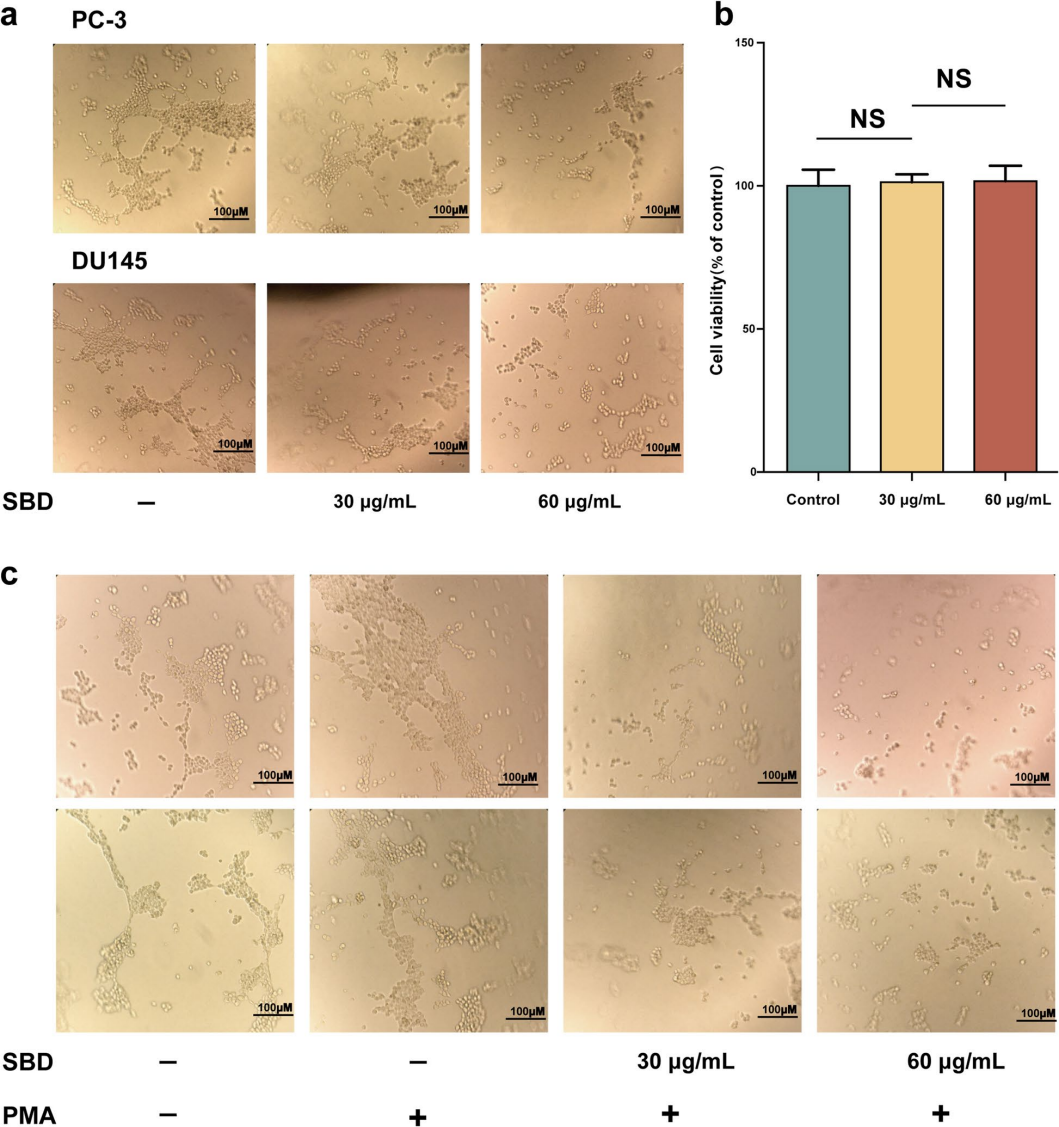
Effects of conditional media (CM) from control- or SBD-treated PCa cells on tube formation, viability of HUVEC. **a** PCa cells were treated without or with SBD at the indicated doses for 48 h. The conditional media (CM) was then harvested and applied to HUVEC cultured in Matrigel-coated plates for an additional 4 h. Changes in cell morphology were captured using an microscope. **b** HUVECs were treated without or with SBD for 4 h, followed by measurement of cell viability by CCK8 (**c**) PCa cells were treated without or with PMA (10 nM), in the absence or presence of SBD for 4 h. The CM was then collected and applied to HUVEC changes in HUVEC tube formation were captured using an inverted microscope. Date was mean ± SEM (*n* = 5), *p* < 0.05 (*)

Angiogenesis is a main process in cancer development and plays a vital role in cancer growth and metastasis. The tumour cells induced by SBD could affect the functions of vascular endothelial cells (HUVECs). HUVECs were incubated with supernatants from SBD-treated PCa cells, and HUVEC tube formation and HUVEC cells viability were measured. There are significance differences between complete media-treated s and CM from PCa cells-treated HUVECs, whilst CM from SBD-treated PCa cells obviously diminished the ability of tube formation of HUVEC in concentration-dependent manner (Fig. 9A). We next investigated whether SBD-mediated inhibition of the tube formation in HUVEC is due to the drug’s cytotoxicity. The viability and survival rate of HUVEC with or without SBD treatment for 48 h did not change significantly, which ruled out the possibility of drug cytotoxicity (*p* > 0.05) (Fig. 9B). It is well known that the PKC activator PMA is able to induce the tube formation of HUVECs cells [22]. When HUVEC were treated with 10 nM PMA, they formed the typical cobblestone morphology characteristic, which was greatly weakened by SBD treatment (Fig. 9C). These results indicated that SBD inhibited angiogenesis by reducing tube formation of vascular endothelial cells rather than apoptosis.

## Discussion

As a global health threat, PCa ranks second in cancer-associated mortality in men in western countries [23]. Currently, the standard treatments for PCa are mainly surgical resection, radiotherapy, and chemotherapy, but the long-term effect of such therapies are still not good, and usually lead to serious side-effects. Nowadays, alternative therapies using Chinese herbal extracts and formulas propose new methods to the administration of anti-PCa [24–26]. Thus, active ingredients in extracts derived from plants have synergistic interactions, which may play a role in fighting against cancer. In recent years, Chinese medicine which possesses excellent bioactivity with low side-effect has been in the clinical application of many diseases. However, despite the accomplishments of researches into finding anti-cancer therapy with Chinese medicines, these medicines are far from being widely applied in the treatment of PCa and there is still a long way to go.

In the current study, we demonstrated that SBD inhibited the growth of PC3 and DU145 in a dose-dependent manner. Clonogenic assay further validated the inhibitory effect of SBD on PCa cells. Meanwhile, we recapitulated the anti-tumour efficacy of SBD *in vivo*. There were no adverse effects in mice during the experiment. These findings implied that SBD had the potential to develop into a safe and effective alternative therapy for PCa patients.

It is well known that he Bcl-2 family proteins are one of the main factors that participate in the apoptosis pathway since they modulate apoptosis by regulating mitochondrial membrane permeability [27]. Bcl-2 is an anti-apoptotic protein, while Bax is an apoptosis inducer [28]. Caspase-3 is the key factor protein as a downstream effector of apoptosis and the increased Caspase-3 level is always used as an indicator of apoptosis [29]. In order to evaluate the molecular mechanism of SBD against PCa, we performed western blot analysis of Cleaved-Caspase-3, Bcl-2 and Bax in PCa cells. Herein, treatment with SBD increased the activities of Cleaved-Caspase-3, enhanced the expression levels of Bax protein, and down-regulated the expression level of Bcl-2 protein, characterizing the extrinsic pathway of cell apoptosis. In addition, the anti-apoptotic function of AKT has been related to inactivation of Bax and Cleaved-caspase-3, as well as overexpression of Bcl-2 [30].

A large amount of evidence indicates that PI3K/Akt signalling pathway is an important regulator involved in cell proliferation and metastasis [31, 32]. Interestingly, we observed the inactivation of PI3K/AKT signalling pathway in PCa cell treated by SBD. The pro-apoptotic and G2/M phase arrest activities of SBD in PCa cells could partially have been caused by the suppression of PI3K/AKT signalling pathway.

EMT is a complex process involving endothelial cell proliferation, differentiation, migration. Increased migration and invasion are actively related to the EMT progression, which is characterized by inhibiting the expression of epithelial markers (E-cadherin and Vimentin) and inducing the expression of mesenchymal markers (N-cadherin) [33–35]. After going through the EMT progress, not only the connection between cells, the contract of cell to matric, but also the normal epithelial polarity disappears among PCa cells. Meanwhile, they gain mesenchymal features to migrate and invade nearby matrix [36, 37].. This process is driven by activating and/or crosstalk between several signalling pathways. Several studies have shown the existence of EMT-related proteins in tumour tissues, especially the EMT marker, an indicator of the poor survival of PCa patients.

Upon activation of PI3K/AKT signalling pathway is disposed to induce the expression levels of EMT-related marker [38]. In addition, the PI3K/AKT signalling pathway is a vital player in EMT in cancer cells [39]. Consequently, our further goal for this investigation was to elucidate potential molecular mechanism of actions for the SBD treatment. Notably, PI3K inhibitor plays a vital role in PCa cell apoptosis and migration by inhibiting the PI3K/AKT signalling pathway [40]. PCa cells were treated with SBD and an PI3K inhibitor (MK2206), followed by incubation for 48 h and the change in cell viability, invasion and EMT were all evaluated. Our results showed that compared with SBD treatment alone, SBD and MK2206 co-treatment have no significant on apoptosis, invasion and the expression of EMT-related protein in PCa cells, which suggested that SBD could inhibit the EMT in PCa cells through attenuating PI3K/AKT signalling pathway.

Antiangiogenic therapy is a critical strategy for the treatment of PCa. Interaction between PCa cells and HUVEC had promoting effects on cell migration between each other, and also contributed to mosaic vessels formation [41]. However, these effects were supressed by SBD treatment. In addition, PMA promoted tube formation of HUVEC, while these promoted effects were also remarkably inhibited by SBD treatment.

## Conclusion

Taken together, our study revealed that SBD could exert the anti-cancer activity in PCa cells, both *in vitro* and *in vivo*. SBD inhibited PCa cells growth, migration and invasion via inducing cell apoptosis, G2/M phase cell cycle arrest, inhibiting EMT progression, which is characterized by attenuating PI3K/AKT signalling pathway. We also exhibit the possible anti-angiogenesis mechanism of SBD in cancer. Accordingly, SBD could play the role as a potential adjuvant agent in treating PCa patients.

## Supplementary Information

**Additional file 1.** Completed “The ARRIVE Guidelines Checklist” for reporting animal data in this manuscript.

**Additional file 2.**

**Additional file 3.**

## Data Availability

The datasets used and analysed during the current study are available from the corresponding author on reasonable request.

## Abbreviations

SBD: Scutellaria barbata D.Don
PCa: Prostate cancer
TCM: Traditional Chinese Medicine;p-AKT, phosphorylated AKT
PI3K: Phosphoinositide 3-kinase
EMT: The epithelial-to-mesenchymal transition
CCK8: Cell counting Kit-8
