# Supplementary material for: Scutellaria barbata D.Don (SBD) extracts suppressed tumor growth, metastasis and angiogenesis in Prostate cancer via PI3K/Akt pathway
Source: BMC Complement Med Ther. 2022 May 3;22:120. doi: 10.1186/s12906-022-03587-0 (PMC9066752; doi:10.1186/s12906-022-03587-0)
Supplement: Supplementary file 2 — Additional file 2. [file 12906_2022_3587_MOESM2_ESM.docx]

Scutellaria barbata D.Don (SBD) extracts suppressed tumor growth, metastasis and angiogenesis in Prostate cancer via PI3K/Akt pathway

Dongya Sheng ^1†^, Bei Zhao ^2†^, Wenjing Zhu ^1†^, Tiantian Wang^1^ and Yu Peng ^1^*

* Yu Peng, Correspondence: [drypeng@sina.com](mailto:drypeng@sina.com)

^1^ Yueyang Hospital of Integrated Traditional Chinese and Western Medicine, Shanghai University of Traditional Chinese Medicine

^2^ Institute of Interdisciplinary Integrative Medicine Research, Shanghai University of Traditional Chinese Medicine, Shanghai, China

Full list of author information is available at the end of the article

^†^The authors make equal contributions and share the first authorship.

Full list of author information is available at the end of the article.


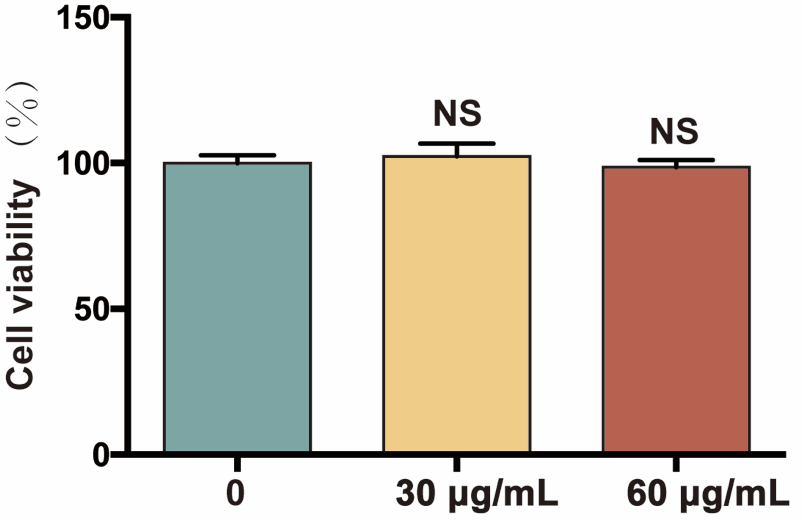


**Figure S1. SBD had no significant effect on wpe-int cells**

Cell viabilities were measured by CCK-8 assay after treatment with indicated concentrations of SBD for 24 h. The cell proliferation and apoptosis of wpe-int cells.
