# Supplementary material for: Scutellaria barbata D.Don (SBD) extracts suppressed tumor growth, metastasis and angiogenesis in Prostate cancer via PI3K/Akt pathway
Source: BMC Complement Med Ther. 2022 May 3;22:120. doi: 10.1186/s12906-022-03587-0 (PMC9066752; doi:10.1186/s12906-022-03587-0)

Scutellaria barbata D.Don (SBD) extracts suppressed tumor growth, metastasis and angiogenesis in Prostate cancer via PI3K/Akt pathway

Dongya Sheng ^1†^, Bei Zhao ^2†^, Wenjing Zhu ^1†^, Tiantian Wang^1^ and Yu Peng ^1^*

* Yu Peng, Correspondence: [drypeng@sina.com](mailto:drypeng@sina.com)

^1^ Yueyang Hospital of Integrated Traditional Chinese and Western Medicine, Shanghai University of Traditional Chinese Medicine

^2^ Institute of Interdisciplinary Integrative Medicine Research, Shanghai University of Traditional Chinese Medicine, Shanghai, China

Full list of author information is available at the end of the article

^†^The authors make equal contributions and share the first authorship.

Full list of author information is available at the end of the article.

**Figure 3c**


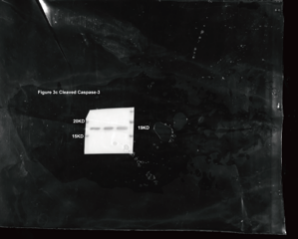

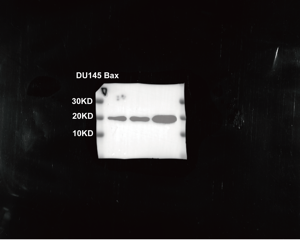

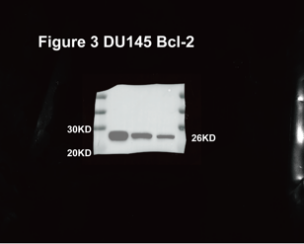


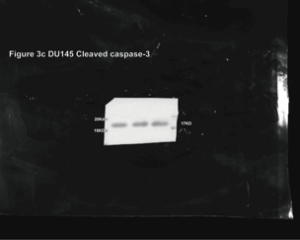

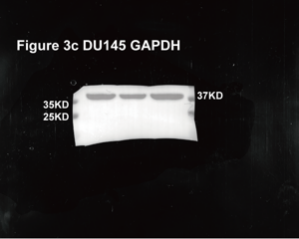

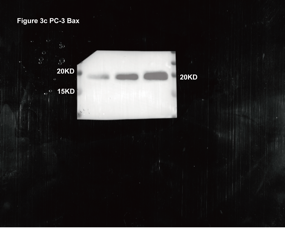

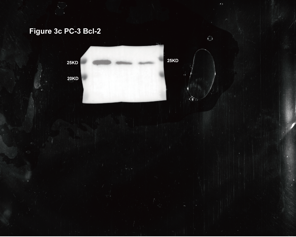

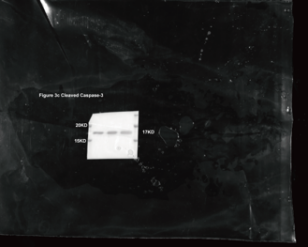

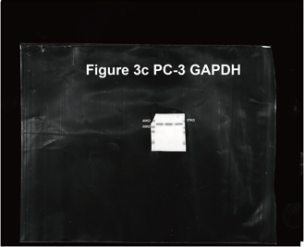


**Figure 3d**


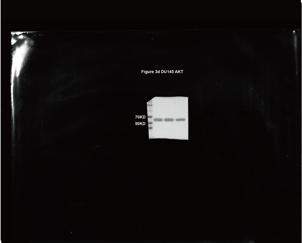

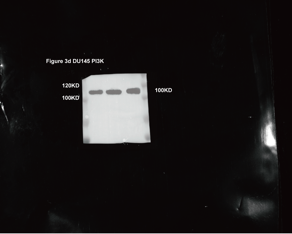

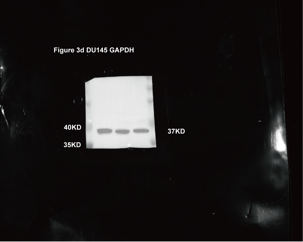

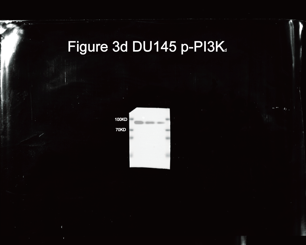

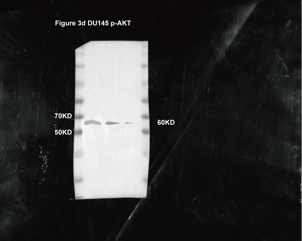

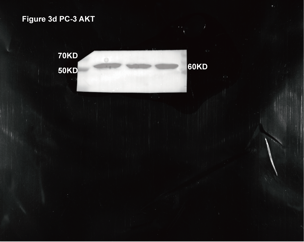

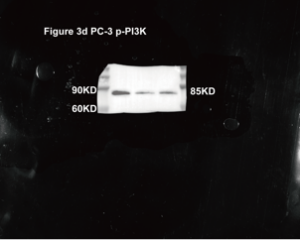

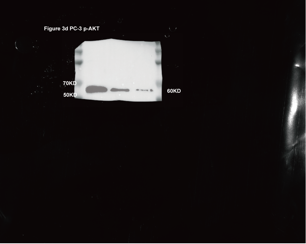

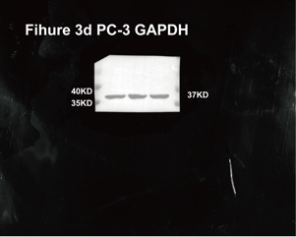

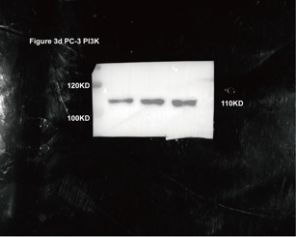


**Figure 4e**

**
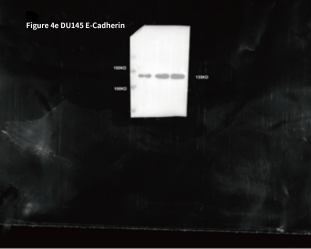

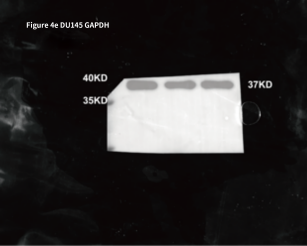

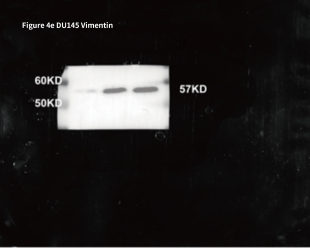

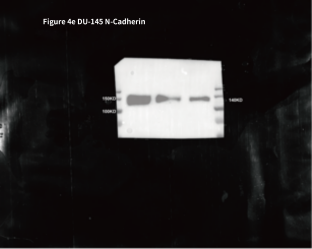

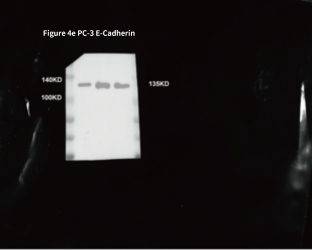

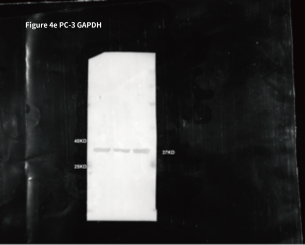

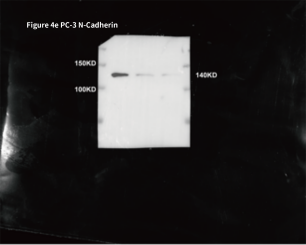

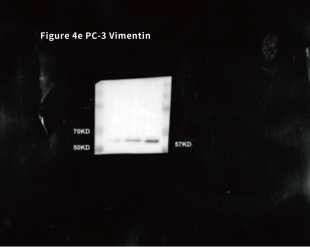
**

**Figure 6a**

**
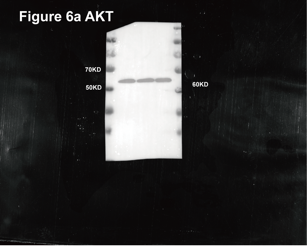

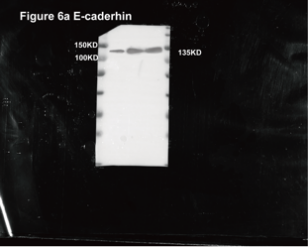

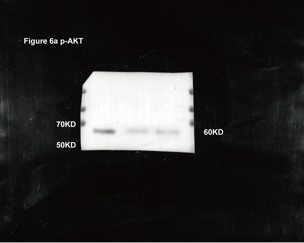

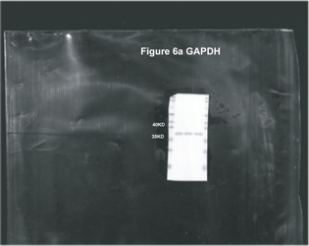

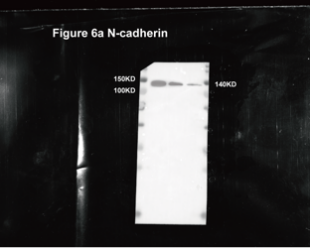

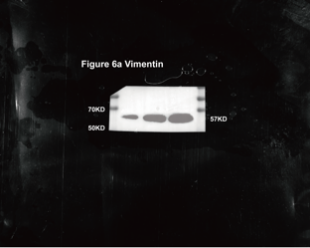
**

**Figure 6b**

**
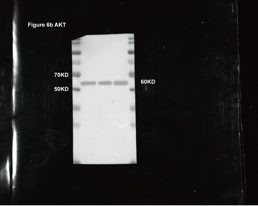

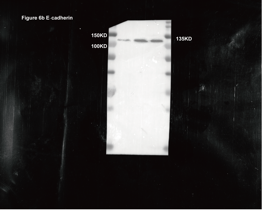

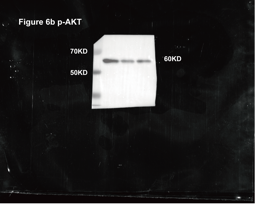

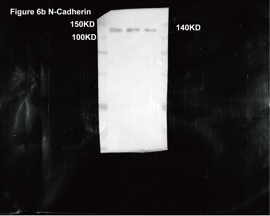

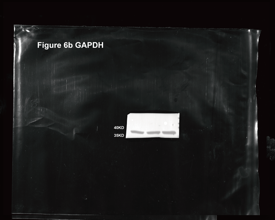

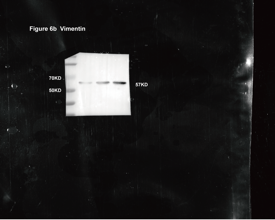
**

**Figure 8a**

**
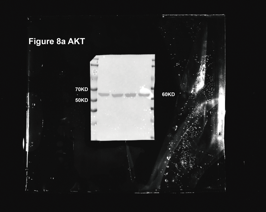

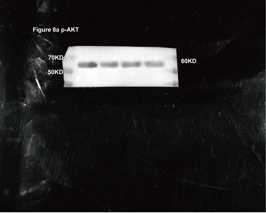

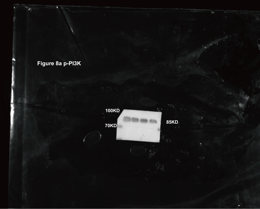

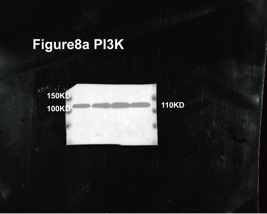
**

one fuller-length, original, unprocessed blot performed with my samples for each antibody which confirms specific detection of the target antigen.

AKT**
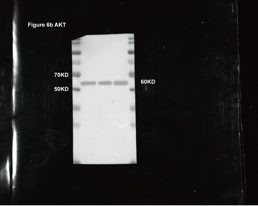
**p-AKT
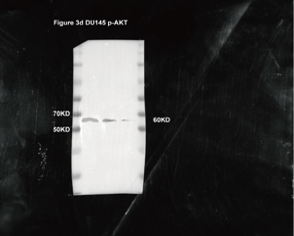
E-cadherin**
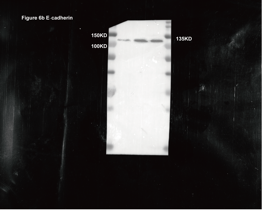
**

N-cadherin**
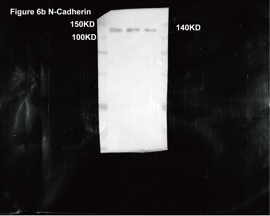
**Vimentin
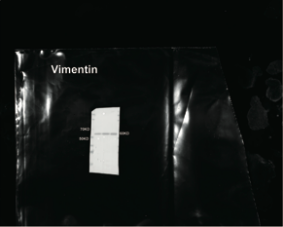
p-PI3K
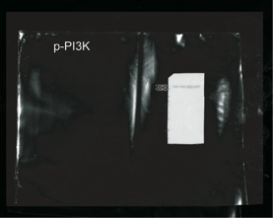


PI3K
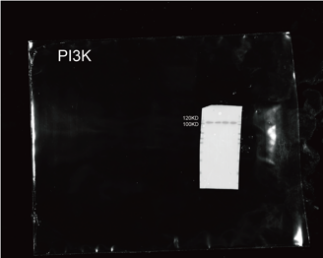
Bax
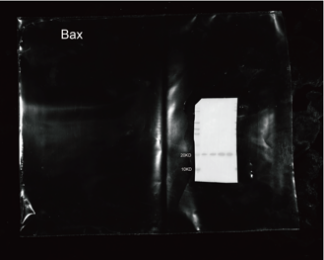
Bcl-2
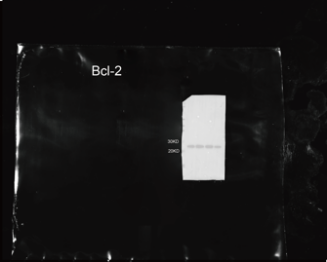


Cleaved caspase-3
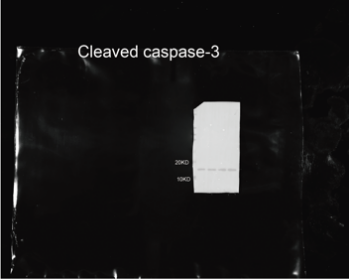

Supplement: Supplementary file 3 — Additional file 3. [file 12906_2022_3587_MOESM3_ESM.docx]
